# Evaluating the Toxic Impacts of Cadmium Selenide Nanoparticles on the Aquatic Plant *Lemna minor*

**DOI:** 10.3390/molecules24030410

**Published:** 2019-01-23

**Authors:** Roshanak Tarrahi, Ali Movafeghi, Alireza Khataee, Farkhondeh Rezanejad, Gholamreza Gohari

**Affiliations:** 1 Department of Plant Biology, Faculty of Natural Sciences, University of Tabriz, Tabriz 51666-16471, Iran; roshanak.tarrahi@gmail.com (R.T.); movafeghi@tabrizu.ac.ir (A.M.); 2 Research Laboratory of Advanced Water and Wastewater Treatment Processes, Department of Applied Chemistry, Faculty of Chemistry, University of Tabriz, Tabriz 51666-16471, Iran; 3 Department of Materials Science and Nanotechnology Engineering, Faculty of Engineering, Near East University, 99138 Nicosia, North Cyprus, Mersin 10, Turkey; 4 Department of Biology, Faculty of sciences, Shahid Bahonar University of Kerman, Kerman 7616913439, Iran; frezanejad@mail.uk.ac.ir; 5 Department of Plant Productions, Medicinal and Aromatic Plants, Faculty of Agriculture, University of Maragheh, Maragheh 55181-83111, Iran; gholamreza.gohari@gmail.com

**Keywords:** nanoparticles, cadmium acetate, cadmium sulfate, *Lemna minor*, toxicity, antioxidants

## Abstract

Cadmium selenide nanoparticles (CdSe NPs) were synthesized by an easy and simple method and their properties were assessed by XRD, TEM and SEM techniques. The effects of CdSe NPs as well as Cd^2+^ ions on *Lemna minor* plants were investigated. The absorption of CdSe NPs by the plants had some adverse consequences that were assessed by a range of biological analyses. The results revealed that both CdSe NPs and the ionic form of cadmium noticeably caused toxicity in *L. minor.* Morphological parameters as well as peroxidase (POD) activity were deteriorated. In contrast, the activities of some other antioxidant enzymes (superoxide dismutase (SOD) and catalase (CAT)) as well as the contents of total phenol and flavonoids went up. Taken all together, it could be implied that CdSe NPs as well as Cd^2+^ were highly toxic to plants and stimulated the plant defense system in order to scavenge produced reactive oxygen species (ROS).

## 1. Introduction

Nanomaterials with at least one dimension of less than 100 nm are basically known as nanoparticles (NPs). The surface to volume (and mass) ratio in nanoparticles is large and potentially causes greater reactivity and motility. As nanomaterials show different properties compared to the bulk materials, potential risks made by their nanometer scale should be taken into consideration. For example, their availability to living organisms is higher due to the smaller size [1]. Nanoparticles exist in the environment, from the natural sources like volcanic activities, wild bushfire, and/or originating from human activities, like smoking, industrial emissions, and technological consumptions [2].

The destiny and distribution pattern of nanoparticles in nature are still unknown and need to be examined. Considerable consumption of nanoparticles in various industries such as electronics, agriculture, medicine, and pharmacy let out abundant wastes, which find their way to ecosystems, including aquatic ones, and harm living organisms in water such as aquatic plants. Therefore, nanotoxicologic studies are focused not only on the physical and chemical characteristics of nanomaterials but also on the health of living organisms impacted by nanoparticles [3]. Nanoparticles are able to go through the cells and induce defense systems. Plants fight against toxicity in different ways including physical responses (such as exclusion the toxic elements through cell wall or their isolation in vacuole) and biochemical reactions (like scavenging reactive oxygen species (ROS) by the activities of antioxidant enzymes and a raise in secondary metabolites contents). But excessive amounts of toxic elements defeat the defense system of plants and reduce germination, growth, and development, and eventually cause death [4,5,6].

Investigations on nanoparticles show that they stick to the surface of cell wall and cell membranes and subsequently penetrate into the cells which cause adverse effects [7]. Furthermore, nanomaterials, to a large extent, release the toxic elements. For example, cadmium selenide nanoparticles contaminate cells both in the forms of nanoparticles, and nanoparticle-released metal ions. CdSe NPs are globally consumed in many industries and have unique mechanical, electrical, optical, and thermal properties, which can be applied in hybrid solar cells, fluorescent imaging, and many other consumptions [8]. Cadmium selenide is a semiconducting material. It has unique optical and chemical properties owing to the quantum size effect. The CdSe NPs have promising applications in many fields, including optics and photo-electrochemical cells. The significant feature relating to these semiconducting nanomaterials is the improvement of the efficient synthesis method. To achieve the optimum properties for these nanomaterials, some manipulations were done. A set of interactions occur among nanoparticles surface and the surrounding liquid in a colloidal solution. The electrolytic interactions, van der Waals forces, solvation forces, and hydrophobic impacts occur to make a sustainable dispersion [8]. Although these nanoparticles have improved life with their extensive applications but the adverse effects caused by their release to the environment should be considered. The cytotoxic impact of Cd^2+^ and many other metallic ions has been widely studied and proved [9]. However, there is little information on the destructive functions of CdSe NPs, especially in plants, and it implies that our knowledge is deficient.

Plants hold the primary position in the food chain of ecosystems and therefore, their contamination by environmental pollutants, for example, nanoparticles, can affect many other organisms linked to the chain [10]. *Lemna minor* is a floating plant in fresh water that grows fast and its application for some studies such as toxicity assessments is easy [11]. The characteristics of *L. minor*, including its small genome size, make it appropriate for toxicological assessments [12].

A facile and economic protocol was applied to synthesize CdSe in nanometer dimensions by hydrothermal route. The features of CdSe NPs were analyzed. Consequently, the harmful impacts of these NPs were evaluated on *L. minor* at various concentrations. The phytotoxic effects were investigated including the changes in antioxidative enzymes activity, total phenol and flavonoid contents, and some physical parameters (e.g., frond size, fresh and dry weights).

## 2. Results and Discussion

### 2.1. Properties of the Synthesized CdSe NPs

**Figure 1:**
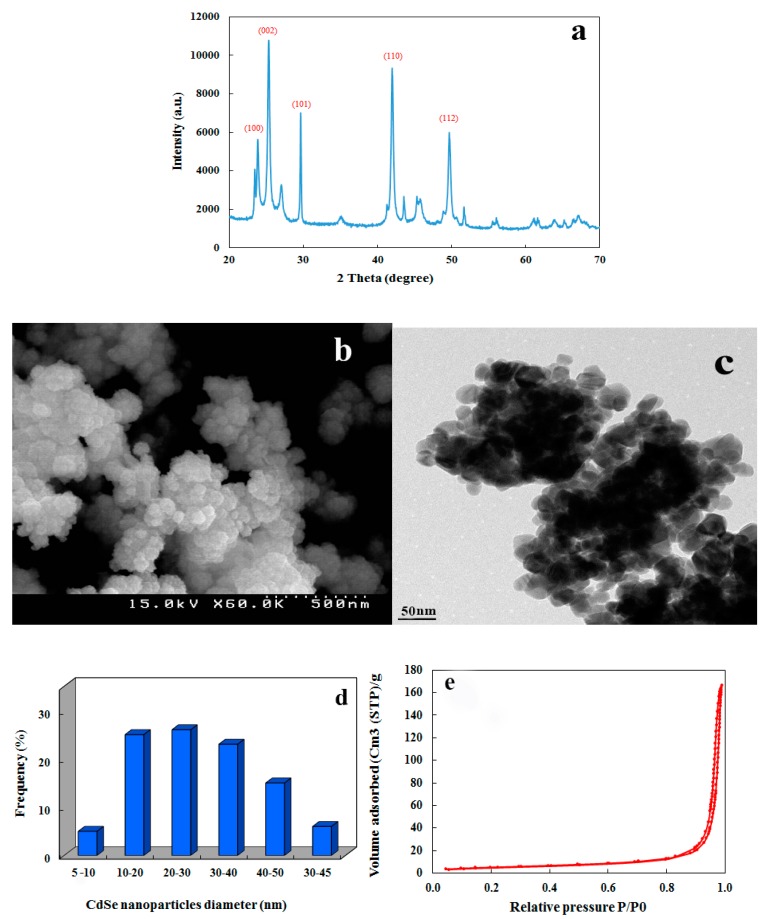
(**a**) XRD pattern of synthesized cadmium selenide nanoparticles (CdSe NPs) with simple hydrothermal method (**b**,**c**) SEM and TEM images of synthesized CdSe NPs (**d**) Various diameter distribution of synthesized CdSe NPs (**e**) N_2_ adsorption-desorption isotherms of CdSe NPs.

X-ray diffraction spectrum of NPs confirmed the synthesis of CdSe NPs (Figure 1a). The diffraction peaks in XRD were at 2θ = 24.32°, 25.4°, 30°, 42.28°, and 49.6° in reference to plane reflections of (100), (002), (101), (110), and (112), and associated with CdSe NPs cubic crystalline 3-dimensional phase (JCPDS 65-2891) (Figure 1a). The sharper (002) peak determined that the nanocrystals were stretched along the c-axis. The XRD pattern confirmed the successful administration of the hydrothermal method in this work. The average crystalline size was 15 nm for CdSe NPs according to Debye–Scherrer formula [13,14]. The SEM micrograph of the synthesized CdSe NPs is shown in Figure 1b. Accordingly, nanoparticles were globe-shaped and their size mostly was in a range between 10 nm to 30 nm (1d). In order to make the morphology of the nanoparticles clearer, the SEM image was prepared (Figure 1b). The high resolution-transmission electron microscopy (HR-TEM) image of CdSe NPs (Figure 1c) proved the acquired results by SEM showing their crystallinity and small size under 100 nm and thus in the range of nanoparticles. Moreover, the surface area and pore size of CdSe NPs were acquired by evaluating the nitrogen adsorption and desorption isotherms (Figure 1e). Consistent with the IUPAC classification, the CdSe NPs samples exhibited type IV isotherms with a hysteresis loop highlighting the mesoporous nature of the samples [15]. The surface area of the sample was 1.7371 E + 01 m^2^·g^−1^ and the pore volume was 0.2569 cm^3^·g^−1^. The CdSe NPs had soft and uniform surface. The mean size of specific surface area was achieved by the following Equation (1) [16]:

D = 6000/Sρ(1)

S demonstrates the surface area of nanoparticles in m^2^ g^−1^ and ρ is density in cm^3^ g^−1^ of CdSe NPs. The NPs size was 13.57 nm that approximately equaled to the crystalline size obtained by XRD. Therefore, the average size of CdSe NPs seemed to be in a range of being up taken by roots of *L. minor*.

### 2.2. Fluorescence Microscopic Imaging

**Figure 2:**
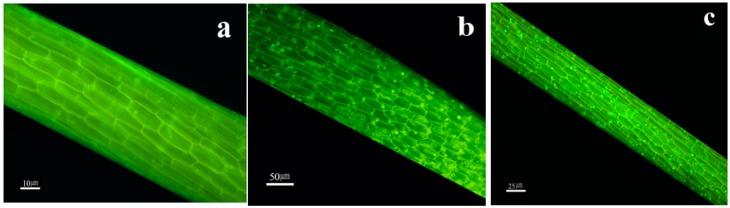
Fluorescence microscopic images of *L. minor* roots (**a**) control, (**b**) treated plants with 40 mg L^−1^ CdSe NPs, (**c**) treated plants with 80 mg·L^−1^ CdSe NPs. Distinguished shiny green marks present nanoparticles.

Fluorescent images of *L. minor* roots, treated by 40 mg L^−1^ and 80 mg L^−1^ of CdSe NPs (average and highest concentrations), were taken by fluorescence microscope and bright green spots were detected as nanoparticles agglomerations in root tissues compared to control with no green stains (Figure 2). Similar fluorescent images of *Spirodela polyrrhiza* roots impacted by l-cysteine capped CdS nanoparticles were formerly introduced [5].

The visible spots of nanoparticles had a direct correlation with their concentration i.e., they were denser and brighter in high concentrations. A key mechanism for eukaryotic cells to embody materials outside the cell is endocytosis. Other routes for NPs to penetrate and pass via cells can be capillary forces, symplastic directions, pores, and plasmodesmata [17]. Properties of CdSe NPs played an important role in their absorbance by the plant [18]. In a study it is reported that fluorescent spots were observed as a consequence of the absorbance of mercaptopropanoic acid coated CdSe/ZNS QDs as well as the entrance of platinum nanoparticles into the cytosol, cell walls and organelles of *Medicago sativa* [19].

### 2.3. Ultrastructure Observation

**Figure 3:**
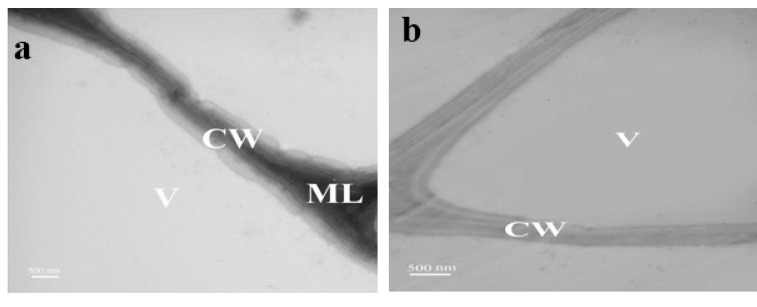

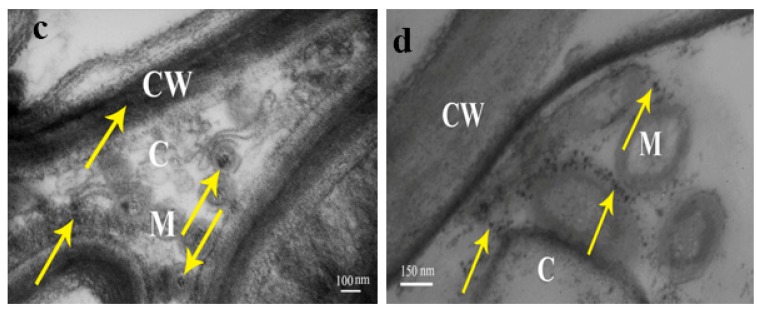

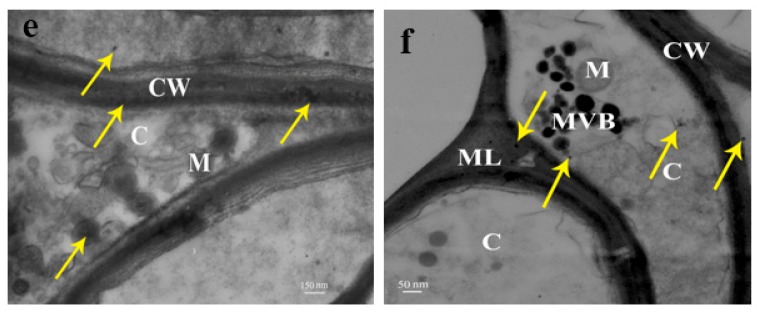
TEM images of *L. minor* root cells. (**a**,**b**) Control comprising cell wall (CW), vacuole (V), and middle lamella (ML). (**c**–**f**) Cells of treated plants with 80 mg·L^−1^ CdSe NPs comprising CW, middle lamella, cytoplasm (C), multiple vesicular bodies (MVB), mitochondria (M), and arrows define CdSe NPs agglomerations.

The entry of NPs in root tissues was assessed and confirmed by TEM (Figure 3). They were penetrated into the cell walls and located inside the cells in the form of aggregations. CdSe NPs could be traced as dark spots (solid arrows) within the cell wall, cytoplasm and multi vesicular structures of the treated plants at the concentration of 80 mg·L^−1^ (Figure 3f). Mitochondria can be seen in Figure 3c–f with degenerated cristae and degraded ER. Also, disrupted organelles of cytoplasm are illustrated in the form of remnants because of destructive impact of CdSe NPs toxicity. Spherical nanoparticles are noticeable in the images. CdSe NPs aggregates are detectable compared to control with no NPs (Figure 3a,b). Eventually these images confirmed CdSe NPs absorbance by *Lemna minor* and their subsequent toxicity. In accordance with the present findings, Au nanoparticles internalization and their destruction was proved by TEM images of rice roots via their entrance through small pores [20]. In addition, the absorption of ZnO NPs in root cells of *Fagopyrum esculentum* has been observed by TEM. Subsequently they had adverse effects and showed phytotoxicity [21]. It is reported that endoplasmic reticulum (ER) stress was concerned to nanoparticles cytotoxicity which consequently caused apoptosis in various cell types e.g., silver nanoparticles induced different ER stress markers in human THP-1 monocytes leading to a rapid ER stress response [22].

### 2.4. Determination of Growth Parameters

**Figure 4:**
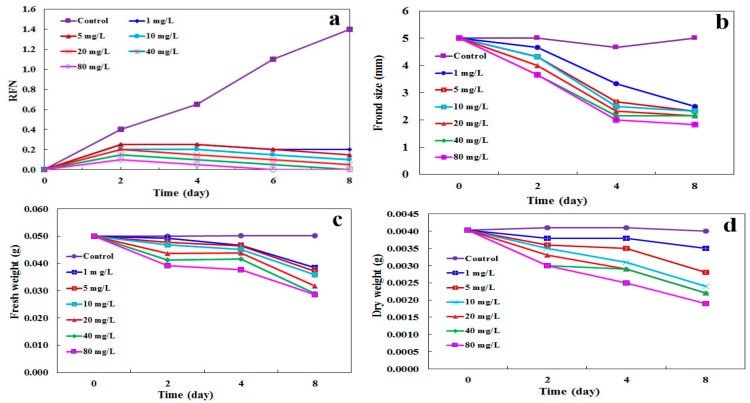
The influence of increasing concentrations of CdSe NPs on the growth parameters of *Lemna minor.* (**a**) Relative frond number (RFN), (**b**) frond size, (**c**) fresh weight, and (**d**) dry weight on the 2nd, 4th, 6th, and 8th days after treatment. Experimental conditions: 1 g of plant in 200 mL of nutrient solution (for each time and concentration treatment), temperature ≈25 °C, pH = 6.5–7, 16/8 (light/dark) photoperiod. The level of confidence is 95% according to Tukey Test (*n* = 3 replicates).

The growth of *L. minor* was analyzed by some morphological factors (growth parameters) such as relative frond number (RFN), frond size, fresh weight, and dry weight to investigate the impact of CdSe NPs. Therefore, the plants were treated with various concentrations of CdSe NPs from 1 to 80 mg·L^−1^ during 8 days (Figure 4). RFN went down significantly in adverse association with the concentration of CdSe NPs and treatment time versus control samples. The concentrations equal and upper than 1 mg·L^−1^ of CdSe NPs poisoned *L. minor* from the second day to the eighth day after treatment (Figure 4a). Keeping on with the growth parameters assessments, *L. minor* evaluations by the means of frond size, fresh and dry weights showed a notable decline during 8 days. Treated plants with CdSe NPs showed a decrease in frond size and fresh weight at 1 mg·L^−1^ and upper on the 4th and 8th day after treatment, and dry weight at 5 mg·L^−1^ and upper from the second day on (Figure 4b–d). Thus, it can be concluded that growth got retarded by nanoparticles due to protein denaturation and photosynthesis inhibition and possibly existence of NPs around vascular bundle prevented water and nutrient intake [21]. These findings are comparable with other works on *L. minor* and *S. polyrrhiza* impressed by ZnO NPs and l-cysteine-capped CdS [5,23], *L. minor* and *Medicago sativa* affected by Ag NPs and CdSe/ZnS QDs respectively [19,24]. Similarly, it was reported that NPs potentially alter the transcription of antioxidant and aquaporin genes hence, unbalance the water absorption in *Arabidopsis* [25]. Possibly, blockage in the function of aquaporins impairs water uptake. Down-regulation of aquaporin genes affected by NPs toxicity has been reported. Thus, the prevention of water uptake, and movement throughout the plant, could be the symptoms of toxicity [26].

### 2.5. Assessing the Activity of Antioxidant Enzymes

As demonstrated in previous works NPs can stimulate ROS production and subsequently oxidative stress, due to their specific characteristics, involving with biological reactions inside the cells [27,28]. As a result, the defense system starts scavenging ROS, but in a case of being too much ROS, disruption in biomolecules and finally cell death occurs [29]. A part of the defense system refers to antioxidant enzymes (e.g., SOD, POD, and CAT) that actively works to remove oxidative stress [30].

**Figure 5:**
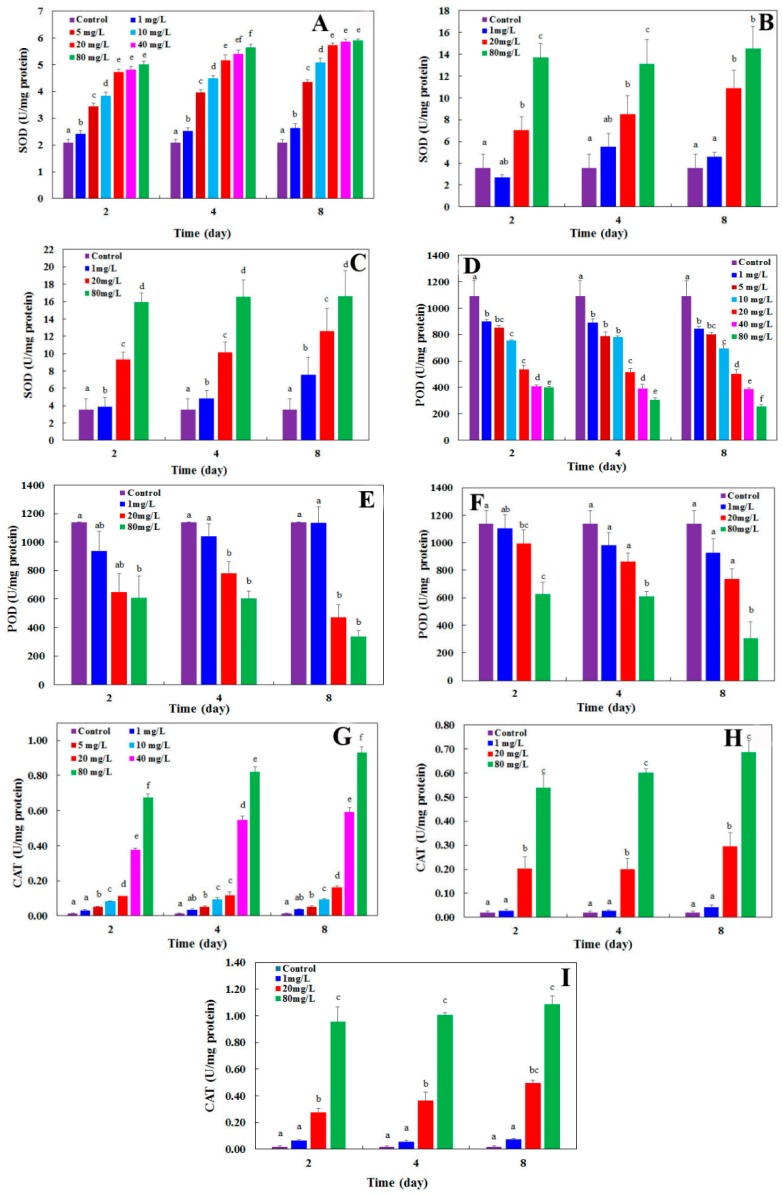
(**A**–**C**) superoxide dismutase (SOD), (**D**–**F**) peroxidase (POD), and (**G**–**I**) catalase (CAT contents in *Lemna minor* influenced by increasing concentrations of CdSe NPs as well as Cd(CH_3_COO)_2_ and CdSO_4_ respectively on the 2nd, 4th, and 8th days after treatment. Different letters indicate significant differences at *p* ≤ 0.05 according to Tukey’s HSD Test. The error bars represent standard deviation of the mean (*n* = 3 replicates).

CdSe NPs effects on antioxidant enzymatic activities, at different concentrations (1 to 80 mg·L^−1^), were investigated in *L. minor* during 8 days. A statistically noticeable change in SOD activity was noted in comparison with the control from 1 mg·L^−1^ of CdSe NPs (*p* < 0.05) and upper at all experimental days (Figure 5a). Also, treated plants with Cd(CH_3_CO_2_)_2_ and CdSO_4_ (Cd^2+^) showed an increase in SOD activity at 20 and 1 mg·L^−1^ and upper (*p* < 0.05) respectively during treatment in comparison with the control (Figure 5b,c). Another study proposed the same results of SOD activity in *L. minor* influenced by CuO NPs as well as Cu^2+^ [31]. SOD is a major ROS hunter that produces H_2_O_2_ and O^2−^, and therefore, the promoted activity of SOD is associated with oxidative stress [32].

POD activity was remarkably reduced in plants treated by ≥1 mg·L^−1^ of CdSe NPs at all experimental days (*p* < 0.05) (Figure 5d). Furthermore, Cd(CH_3_CO_2_)_2_ poisoned plants and suppressed POD at ≥20 mg·L^−1^ concentrations (*p* < 0.05) at all treatment days (Figure 5e). CdSO_4_ impacted on plants from 1 mg·L^−1^ from day 4 after treatment (Figure 5f).

The reduction in POD activity, at concentrations upper and equal to 1 mg·L^−1^ (*p* < 0.05), could because of elevated ROS introduction influenced by CdSe NPs that very likely denatured enzyme structure. The results for POD activity showed that adverse effect of nanoparticles was stronger than the ionic forms of cadmium. In the other work similar outcomes were reached for POD content in onion plants affected by TiO_2_ NPs [33].

A significant increase was noted in CAT content at >5 mg·L^−1^ of CdSe NPs (Figure 5g) (*p* < 0.05) and at ≥20 mg·L^−1^ of Cd(CH_3_CO_2_)_2_ and CdSO_4_ (Figure 5h,i) during all experimental days. It could be concluded that ROS initiation is stimulated even in low concentrations of CdSe NPs. Also, the ionic form of cadmium (Cd^2+^) was toxic to the plant and higher concentrations provoked more toxicity compared to the NPs. There are some results in consistent with our work that CAT activity has been elevated in *L. minor* influenced by CuO NPs in addition to Cu^2+^ [34]. CAT is an important antioxidant enzyme and converts H_2_O_2_ to water and oxygen. CAT usually follows the same direction as SOD under stress [32].

With the obtained results, showing an increase in SOD and CAT activities in contrast to POD with a decrease, it can be concluded that ROS generation has been dominated over its scavenging and therefore, the NPs led to oxidative stress in the plant.

Altogether, ions can catalyze the generation of ROS; also NPs are able to produce ROS and oxidative stress, in addition to free ions and mitochondrial deterioration [35]. The toxicity function of Cd ions was possibly due to the binding of metal to the sulfhydryl groups in proteins that caused destruction or prevention in proteins activity [36]. Enzymes are the key targets of heavy metal ions [37]. Also, cadmium could affect the availability of minerals that were needed to be up taken by plants [38]. Furthermore, stomatal opening, transpiration and photosynthesis got influenced by cadmium [39].

### 2.6. Evaluation of Total Phenol, Flavonoids, and Malondialdehyde (MDA) Content

The flavonoids and phenols quantity is modified by various stresses [40]. Phenolic compounds participate in ROS scavenging possibly as reducing agents, hydrogen contributors and singlet oxygen suppressors [41]. Similarly, flavonoids contribute to stress resistance against stressors like heavy metals as well. Information on total phenolic compounds, in plants against NPs is insufficient. They consist aromatic benzene ring compounds with chelating capability against stress [42].

**Figure 6:**
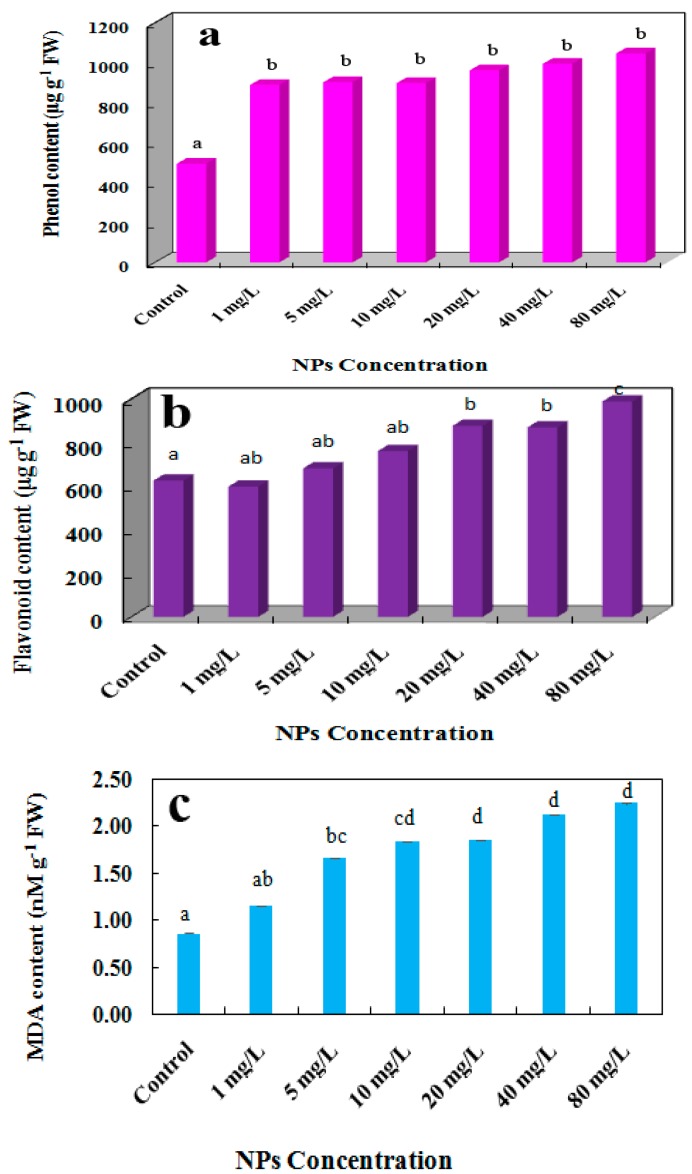
Impact of CdSe NPs on total (**a**) phenol, (**b**) flavonoid, and (**c**) MDA content, respectively. The differences are significant. The level of significance is determined at *p* ≤ 0.05 according to Tukey Test (*n* = 3 replicates).

The content of total phenol grew significantly in plants influenced by CdSe NPs from 1 to 80 mg L^−1^ in a dose and time dependent manner in comparison to control which was started from 888.85 μg·g^−1^ FW and reached to 1046.60 μg·g^−1^ FW of phenol at the maximum concentration of NPs (Figure 6a). The volume of flavonoids was raised in the plants treated with CdSe NPs from 1 to 80 mg·L^−1^ during 8 days as well. The flavonoid quantities started from 681.58 μg·g^−1^ FW and went up to 988.35 μg·g^−1^ FW (Figure 6b). Similar results were reported on *Brassica nigra* impacted by ZnO NPs in nonenzymic antioxidative compounds including phenol and flavonoid contents [43]. The imbalance between produced ROS and scavengers as antioxidant enzymes has been insufficient for removing oxidative stress. Thus, this rise could be associated with toxicity which activated another part of the defense mechanism in cells to help the enzymatic function and promote the whole defense mechanism.

The initiation of lipid peroxidation caused by oxidative stress can be computed based on the commencement of MDA which is an indicator of lipid peroxidation. Lipid peroxidation caused by a rush of ROS, leads to cell membrane decomposition. Hence, alteration in MDA content is dependent on ROS accumulation [44]. Thus, MDA volume was measured in *L. minor* to assess membrane integrity impacted by CdSe NPs (1–80 mg·L^−1^).

The MDA content of the plant went up statistically significant in a dose and time dependent manner. In treated plants with 1 mg·L^−1^ to 80 mg·L^−1^ of CdSe NPs it commenced from 1.15 nM·g^−1^ and went up to 2.24 nM·g^−1^ (Figure 6c). This gradual grow could contribute to the existence of superoxide radicals produced from lipid peroxidation [44]. There are some studies in agreement with the present work, for example, in one work it is shown that elevated quantity of MDA was influenced by CeO_2_ NPs in lettuce [45].

## 3. Materials and Methods

### 3.1. Plant Collection, Culture, and Exposure Conditions

*L. minor* plants were collected from a small lake in Alijan near Bostanabad, 80 kilometers from Tabriz, Iran. They were sterilized in the laboratory by 5% sodium hypochlorite for 5 min and were fed with 30% Steinberg culture medium. This solution contained 349 mg·L^−1^ KNO_3_, 90 mg·L^−1^ KH_2_PO_4_, 12.5 mg·L^−1^ K_2_HPO_4_, 108 mg·L^−1^ Ca(NO_3_)_2_·4H_2_O, 49 mg·L^−1^ MgSO_4_·7H_2_O, 0.04 mg·L^−1^ NaMoO_4_·2H_2_O, 0.456 mg·L^−1^ FeCl_3_·7H_2_O, 0.1 mg·L^−1^ MnCl_2_·2H_2_O, 1.1 mg·L^−1^ H_3_BO_3_, 0.1 mg·L^−1^ ZnSO_4_·7H_2_O, and 1.5 Mg·L^−1^ Na_2_EDTA.

The room temperature and photoperiod were regulated at 20–25 °C and 16/8 h light/dark, respectively. The culture medium containing a nourishing mixture was replaced almost every week for the optimum growth of plants.

To set up the experimental design, a search was performed to find toxicity levels in *L. minor.* The experiences were repeated 3 times for both the control and treated samples with 1, 5, 10, 20, 40, and 80 mg·L^−1^ of CdSe NPs. Various concentrations of nanoparticles were obtained using a stock with 1000 mg·L^−1^ of CdSe nanoparticles. The stock solution was sonicated for half an hour prior to use. Depending on the tests, different exposure times were applied, during a week by two-day intervals, as presented in each part. Also, different concentrations depending on the purpose of the test were used, e.g., medium and maximum concentrations (40 and 80 mg·L^−1^ of CdSe NPs) for fluorescence microscopy observation showing the effect of concentration on the entrance. To demonstrate the effect of various concentrations on biochemical and physical properties a range between 1 to 80 mg·L^−1^ of NPs were used (from the lowest to the highest toxicity). Moreover, based on the analysis, the plants were picked by their weight (e.g., for the assessment of antioxidant activities or MDA, flavonoids, and phenols contents with 250 mg for each test and repeated 3 times) or by number (e.g., for RFN and frond size with 20 isometric fronds and for fresh and dry weights with 40 isometric fronds).

### 3.2. CdSe NPs Synthesis

The following materials: Cd(CH_3_COO)_2_·2H_2_O 98%, N_2_H_4_·H_2_O 80%, Na_2_SeO_3_ 99% (LobaChemie Co., Mumbai, India), NaOH (Merck Co., Darmstadt, Germany), C_10_H_14_N_2_O_8_Na_2_·2H_2_O (Rankem Co., Haryana, India), and C_2_H_6_O 90% (Iran Daru Co., Tehran, Iran) were provided in analytical grade.

Hydrothermal method was used for preparing CdSe nanoparticles as follows: first 1 mmol Na_2_SeO_3_, 1 mmol NaOH and Cd(CH_3_COO)_2_·2H_2_O were mixed with 70 mL distilled water. Then, 1 mmol EDTA was dissolved in 20 mL distilled water at an average stirring pace and it was added to the first solution. Under stirring, hydrazine was added drop wise as a reducing agent. Then, the ultrasonic was applied for 20 min (Sonica, 2200 EP S3, SOLTEC, Milan, Italy) and a 150 mL Teflon-coated stainless-steel was used for autoclaving at 180 °C for 24 h. Then, it was cooled to the ambient temperature. The black deposit was rinsed with distilled water and ethanol to wash away all impurities. The sediment was dehydrated at 60 °C for 5 h.

### 3.3. CdSe Nanoparticles Properties

CdSe NPs were analyzed by XRD, SEM, TEM, and the Brunauer, Emmett, and Teller (BET) techniques. XRD spectrum was recorded in ambient temperature to ascertain the crystalline phase of the CdSe NPs. To fulfill this task, D8 Advance diffractometer device (Bruker, Hamburg, Germany) was used with Cu Kα radiation (λ = 1.5406 A°). The morphological features of nanoparticles were determined by SEM (S-4200, Hitachi, Ibaraki, Japan). In addition, high-resolution transmission electron microscopy (HR-TEM) pictures of synthesized CdSe NPs were acquired by a Cs-corrected high resolution TEM (JEM-2200FS, JEOL, Tokyo, Japan) operating at 200 kV. A BET test was applied by a Belsorp mini II instrument utilizing nitrogen adsorption/desorption at 77 K (Bel, Osaka, Japan).

### 3.4. Epifluorescence Microscopy

Epifluorescence microscopy was used for CdSe NPs detection inside the roots of treated plants. The observation was done a week after the treatment of *L. minor* by CdSe NPs in comparison to control. The roots were sectioned and merged in 0.1% Auramine O for 10 min. The specimens were assessed precisely under Olympus BX51 fluorescence microscope supplied by fluorescence illuminator. The wavelengths were adjusted to 480 to 510 nm (Olympus Optical Co., Ltd. Tokyo, Japan). Stack z-projection could give the best images for the eventual setting of images [46].

### 3.5. Transmission Electron Microscopy

Transmission electron microscopy (TEM) was used for cellular ultrastructure detection. The roots of plants were exposed to CdSe NPs for one week and then were cut and prefixed in a fixative containing 2% (*v*/*v*) glutaraldehyde in 100 mM phosphate buffer. The pH was set on 7.4 and the solution was kept for 24 h at 4 °C. Phosphate buffer was used to wash the samples for four times. Afterwards, they were post fixed with 2% (*w*/*v*) osmium tetroxide for 2 h. Ethanol series were applied to dehydrate the specimen and then immersed in araldite epoxy resin. The thickness of samples was 60 nm applied by Leica ultramicrotome with a diamond knife (Leica Mikrosysteme, A-1170, Wien, Austria). At last, uranyl acetate dissolved in methanol, in addition to Reynold’s lead citrate, were applied for staining the samples [47]. Observation was performed by LEO 906 TEM at 80 kV electron voltage.

### 3.6. Assessments of Morphological Parameters

Relative frond number (RFN), frond size, fresh weight, and dry weight of *L. minor* were assessed as growth factors when encountering CdSe NPs from 1 to 80 mg·L^−1^ in comparison to control. For RFN and frond size calculation, *L. minor* plants with 20 relative isometric fronds were treated by different concentrations of CdSe NPs. Fresh and dry weight analyses were executed at the same concentrations with 40 relative identical fronds of plants. All tests were done under natural light and room temperature in the laboratory.

The growth rate of plants, including mentioned tests was conducted by a stereomicroscope (Olympus, Japan) and digital scale (Adam equipment, AAA 250L, USA) at 2-day intervals during 8 days. The RFN was measured using Equation (2) [48].

RFN = frond (N_1_) − frond (N_0_)/frond (N_0_) (2)

N_0_ and N_1_ show frond numbers at day 0 and day N in turn.

### 3.7. Evaluation of Antioxidative Enzymes

CdSe NPs (1 to 80 mg·L^−1^) along with ionic forms of cadmium including cadmium acetate (Cd(CH_3_CO_2_)_2_) and cadmium sulfate (CdSO_4_) (at 1, 20 and 80 mg·L^−1^ as minimum, medium, and maximum concentrations) were utilized to treat *L. minor* for calculating enzymes activities versus control every 2 days during a week. The extract of fresh plants (0.250 g) was obtained by extraction buffer (0.1 mol, pH = 7.0) having 2% PVP (*w*/*v*). After centrifugation for 15 min (6000× *g*) at 4 °C, the supernatant was consumed for the test [49]. The measurements were done in quadruplet.

The activity of SOD (EC 1.15.1.1) was measured by Beyer and Fridovich method [50]. The SOD solution was prepared with 2.65 mL potassium phosphate buffer solution (67 mmol·L^−1^, pH = 7.8), 0.1 mL NBT (1.5 mmol·L^−1^), 0.2 mL EDTA (0.1 mmol·L^−1^) including 0.3 mmol·L^−1^ KCN, 50 μL riboflavin (0.12 mmol·L^−1^), and 50 μL enzyme extract. The mixture was put under 1000 Lux light intensity for 15 min. The absorbance was read at 560 nm. One unit of SOD equals to the volume of enzyme that restrains 50% of the NBT depletion under the analysis state [51].

POD (EC 1.11.1.7) activity was prepared based on the technique of Chance and Maehly’s. The solution was prepared using citrate-phosphate-borate buffer solution (0.1 mol·L^−1^, pH = 7), 25 μL enzyme extract, 15 mmol·L^−1^ guaiacol and 3.3 mmol·L^−1^ H_2_O_2_. The absorbance was recorded at 470 nm for 3 min as the guaiacol was polymerized. One unit of POD activity was considered as the quantity of enzyme that could engender 1 µmol·L^−1^ tetraguaiacol min^−1^ [ε = 26.6 (mmol·L^−1^)^−1^·cm^−1^]. The amount of enzyme was calculated regarding to a mg of protein [52].

The activity of CAT (E.C. 1.11.1.6) was assessed according to the Chance and Maehly’s method. The mixture comprised 25 µL enzyme extract, citrate phosphate-borate buffer solution (0.1 mol·L^−1^, pH = 7.5) and 10 mmol·L^−1^ H_2_O_2_. The absorbance was read at 240 nm [ε = 39.4 (mol·L^−1^)^−1^·cm^−1^] through 3 min. One unit of CAT content was calculated as the quantity of enzyme that reduced 1 µmol of H_2_O_2_ each minute. The amount of enzyme was assigned regarding to a mg of protein [52].

The Bradford assay was used for determining protein concentration by a UV-Vis spectrophotometer (WPA light wave S2000, England) with bovine serum albumin as the standard reference [49].

### 3.8. Evaluation of Nonenzymatic Antioxidative Compounds

The content of phenolics was measured using modified method of singleton et al., as follows: An amount of 100 μL of the plant extract (1 mg·mL^−1^ methanolic solution of the extract) was blended with 100 μL Folin–Ciocalteu’s reagent in 2.5 mL water for 6 min and 150 μL NaHCO_3_ (20%). After keeping the mixture for 30 min at ambient temperature, the absorbance was recorded at 765 nm by UV/Vis spectrophotometer. Gallic acid was applied as a standard for calibration line with the same approach. The quantity of phenolics was acquired (μg·mL^−1^) by measuring the absorbance based on calibration line equivalent to the gallic acid (μg of GA g^−1^ of extract) [53].

Flavonoid content was calculated based on the method previously reported [54]. The plant was extracted in methanol (1 mg·mL^−1^) and blended with 2% AlCl_3_ methanol solution (1 mL of each). After an hour, the absorbance was recorded at 415 nm. A calibration line was parallel to the standard mixture. The flavonoid’s concentration was analyzed (μg·mL^−1^) based on the absorbance acquired by calibration line and eventually were presented concerning the quercetin equivalent (μg of QE g^−1^ of extract). The analyses were repeated three times.

Thiobarbituric acid-malondialdehyde (TBA-MDA) content was achieved based on a previously reported method [55]. The plant (0.1 g FW) was extracted in 2 mL trichloroacetic acid 0.1% (TCA) and centrifuged (13,000× *g*) for 15 min. 250 µL of the supernatant was mixed with 2 mL 0.5% TBA reagent including 20.0% (*w*/*v*) trichloroacetic acid, kept in a block heater bath (Fater Electric, w 350 B, Tehran Iran) for half an hour at 95 °C, cooled immediately and centrifuged (13,000× *g*) for 10 min. The absorbance was read at λ_max_ = 440, 532, and 600 nm. For the control, 250 µL TCA instead of plant extract was used with the same procedure. To compute MDA content, extinction coefficient was 156 mM^−1^·cm^−1^ in the following Equation (3):

LP (nmol·ml^−1^) = (A_532_ − A_600_) − (A_440_ − A_600_) (MA of sucrose at 532 nm)/(MA of sucrose at 440 nm) × 10^5^ (3)

Molar adsorption (MA) of 1–10 mM sucrose at 532 and 440 nm are 8.4 and 147, respectively that corresponds to 0.0571. MDA content is expressed as nM MDA g^−1^ FW.

### 3.9. Statistical Analysis

The obtained data was analyzed applying GraphPad Instat 3 software (GraphPad software, San Diego, CA, USA). One-way analysis of variance using multiple comparison tests based on Tukey were performed in triplets. Statistical significance was considered at *p* ≤ 0.05.

## 4. Conclusions

Treated *L. minor* with CdSe NPs showed acute toxicity. In spite of the stimulated defense system, severe impacts suppressed the plants. The results showed that CdSe NPs invasion decreased growth parameters but raised MDA and total phenol and flavonoid content, in addition to SOD and CAT activities (two antioxidant enzymes). In contrast, POD activity (another antioxidant enzyme) was decreased significantly, which might be due to denaturation in the enzyme structure. Furthermore, the application of cadmium acetate and cadmium sulfate led to less severe toxicity in *L. minor.* As a result, CdSe NPs provoked poisoning due to their specific properties in addition to released ions. The exposed plants started ROS accumulation that subsequently turned defense system of the plant on for its survival.
